# Fabrication of a Flexible Lab-Made PVP-CuO Modified Electrochemical Sensor for Dual Detection of Dopamine and Paracetamol

**DOI:** 10.1021/acsomega.6c01227

**Published:** 2026-04-27

**Authors:** Duygu Zabi̇tler, Esra Ülker, Gözde Aydoğdu Tığ

**Affiliations:** † 37504 Ankara University Graduate School of Natural and Applied Sciences, Dışkapı, Ankara 06110, Turkey; ‡ Faculty of Science, Department of Chemistry, Ankara University, 06100 Ankara, Turkey

## Abstract

Flexible screen-printed electrodes (FSPEs) fabricated from eco-friendly and recyclable materials represent a promising strategy for developing cost-effective and sustainable electrochemical sensors. In this study, FSPEs were fabricated on polyethylene terephthalate (PET) substrates obtained from recycled plastic bottles, using carbon and silver conductive inks, with colorless nail polish as an insulating layer. To enhance electrochemical performance, the working electrode surface was modified with a polyvinylpyrrolidone-copper oxide (PVP-CuO) nanocomposite via drop casting. The PVP matrix ensured uniform film formation and improved electrode stability, while CuO nanoparticles provided abundant electroactive sites and promoted faster electron transfer. The fabricated electrode displayed notable electrocatalytic activity for the simultaneous determination of dopamine (DA) and paracetamol (PAR), two analytes commonly present in biological fluids and pharmaceutical formulations. In addition, the PVP/CuO modification significantly improved the electrode’s sensitivity and selectivity toward both analytes by facilitating efficient analyte–electrode interactions and enhancing charge-transfer kinetics. Under optimized conditions, the PVP/CuO modified FSPE sensor demonstrated low detection limits of 1.188 μM for DA and 1.024 μM for PAR, with a wide linear range of 4.0–1000.0 μM and good reproducibility. The proposed platform offers a sustainable, low-cost, and efficient approach for the simultaneous electrochemical detection of DA and PAR.

## Graphical abstract

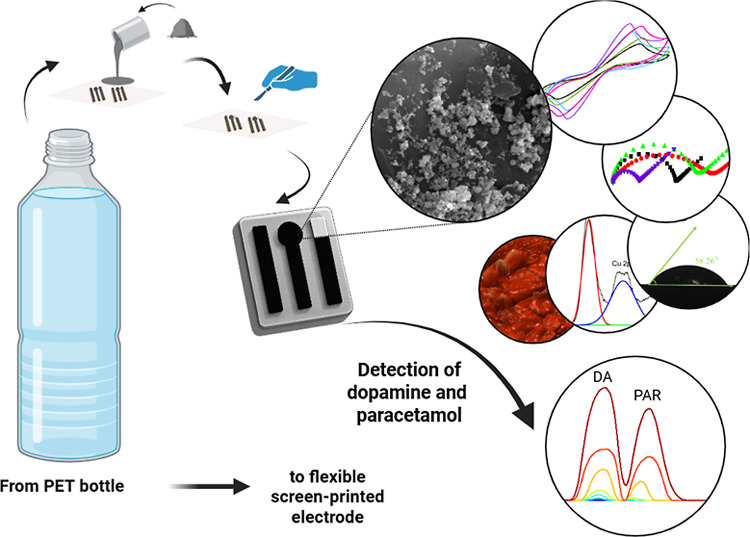

## 1 Introduction

Technological advances have precipitated a marked increase in demand for low-cost, portable, and fast-response sensor systems. In this context, electrochemical sensors have attracted considerable attention in clinical, industrial and environmental analyses due to their simple fabrication, low cost, and wide applicability. Among these, screen-printed electrodes (SPEs) have become an essential component of electrochemical analysis because of their portability, sensitivity, and suitability for various applications.^1,2^ However, most SPEs are fabricated on rigid substrates, such as ceramics, which limits the flexibility of the electrodes. Consequently, alternative substrates such as PET and paper have been explored, offering advantages including low cost, miniaturization, disposability, environmental sustainability, and improved flexibility.^2–5^

The fabrication of flexible screen-printed electrodes (FSPEs) in a laboratory setting involves a series of technical steps that can be executed using relatively straightforward equipment. The initial step in this process is designing electrode patterns to match the desired geometry. This is followed by preparing conductive inks compatible with the selected substrate. Common substrates include polyethylene terephthalate (PET), paper, and other polymeric films, which are selected for their flexibility, chemical stability, and cost-effectiveness.^6–10^ Among these materials, PET is widely used due to its mechanical strength, chemical resistance, low water absorption, and recyclability, making it a reliable and environmentally friendly substrate for flexible electrochemical sensors.^11^

Notable drawbacks of commercially available SPEs include high costs averaging about 5.00 USD per unit, limited customization, and technical constraints in meeting specific experimental needs.^12^ This study focused on producing customizable SPEs from recycled PET bottles to address both economic and environmental limitations. As shown in Table S1, the laboratory manufacturing cost of a single modified-FSPE (MFSPE) is 0.4565 USD, which is roughly 11 times lower than that of commercial alternatives. This cost advantage highlights the potential of MFSPEs for budget-limited research and low-cost sensor applications.^4,10^ In this context, this study aimed to develop a sustainable, cost-effective electrochemical platform by reevaluating a waste material.

Paracetamol (acetaminophen, *N*-acetyl-*p*-aminophenol, PAR) is a widely used nonopioid analgesic and antipyretic incorporated into both prescription and over-the-counter medications.^13,14^ It is commonly prescribed for the treatment of headaches, toothaches, arthritis, and postoperative pain and is generally considered safe within the recommended dosage range.^15,16^ However, prolonged or excessive administration may lead to hepatotoxicity and nephrotoxicity due to the accumulation of reactive metabolites, particularly *N*-acetyl-*p*-benzoquinone imine.^15^ Moreover, increased consumption during the COVID-19 pandemic has resulted in its frequent detection in wastewater, raising ecological concerns and producing toxic chlorination byproducts. Notably, the electroactivity of paracetamol through oxidation of its phenolic group enables sensitive and rapid electrochemical determination even in complex matrices.^17^ Therefore, reliable determination of paracetamol in pharmaceutical and biological samples remains of considerable importance.^14,16^

Dopamine (DA) is an electroactive catecholamine neurotransmitter functioning as a biogenic amine in the brain, kidneys, and peripheral systems.^18–20^ In the central nervous system, it regulates essential cognitive and physiological processes such as mood, behavior, attention, stress, and memory.^16,21^ Abnormal variations in DA levels are closely linked to a variety of conditions affecting both the central nervous system and mental health, such as Parkinson’s disease, Alzheimer’s disease, schizophrenia, fibromyalgia, depressive disorders, restless legs syndrome, and burning mouth syndrome.^15,22^ In Parkinson’s disease, degeneration of dopaminergic neurons reduces dopamine levels in the striatum and basal ganglia^16^ whereas substance abuse (e.g., heroin, alcohol, nicotine, and cocaine) can cause pathological increases in DA levels, contributing to cardiovascular complications.^15^ Owing to its oxidizable structure, dopamine can be rapidly and sensitively quantified using electrochemical techniques, which are also suitable for colored and complex biological samples.

DA and PAR are physiologically relevant electroactive species that may coexist in biological systems, with their bioavailability often mutually influencing each other. Accurate quantification of these compounds is therefore essential for effective therapeutic management, monitoring potential drug interactions, and ensuring appropriate dosage adjustments. However, their oxidation through phenolic hydroxyl groups occurs at similar potentials, leading to overlapping voltammetric responses on conventional electrodes and hindering selective detection. Consequently, rapid, portable, reliable, and highly sensitive electrochemical techniques are required for the precise and selective determination of DA and PAR in complex biological matrices.^15,16^

Metal oxide nanoparticles offer considerable potential for electrochemical sensing applications due to their unique chemical and physical properties.^23,24^ Among metal oxide NPs, the copper oxide (CuO) has a low band gap (∼1.2 eV), a high surface area, electrical conductivity, and antibacterial/anticancer potential.^25–28^ However, CuO nanoparticles have a strong tendency to aggregate due to their high surface energy, thereby reducing their stability and limiting their analytical performance.^25,29^ To overcome this limitation, polymer coatings such as polyvinylpyrrolidone (PVP) are frequently employed to stabilize metal nanoparticles due to their hydrophilicity, chemical stability, biocompatibility, and low toxicity.^30^ The pyrrolidone groups of PVP interact strongly with the CuO surface through hydrogen bonding and acid–base coordination between the carbonyl groups of PVP and the surface hydroxyl sites of CuO nanoparticles, preventing agglomeration and maintaining colloidal stability while promoting more uniform dispersion and improved electron-transfer pathways.^25,31–33^ In addition, the nonionic and neutral nature of PVP minimizes background signals in electrochemical measurements, thereby enhancing analytical accuracy and selectivity. The electrocatalytic properties of CuO nanoparticles further contribute to the selective detection of PAR and DA.^34^ Accordingly, PVP-stabilized CuO nanoparticles were used to modify the electrode surface, providing a stable, electroactive interface.^25,31^

In the literature, PET-based substrates have been widely used in disposable electrochemical sensors as replaceable, nonreplaceable, or modified working electrodes. For example, Andreotti et al. reported a single-use electrochemical sensor fabricated from PET, nail polish, and graphite powder^35^ while Ferreira et al. developed a PET-based disposable carbon-ink SPE immunosensor for SARS-CoV-2 detection.^36^ Similarly, Ülker et al. described a PET-based disposable electrode composed of graphite and nail polish for the simultaneous detection of acetaminophen and caffeine.^3^ Inspired by these studies, a PET substrate was employed in this work, and nail polish was selected as the binder for the graphite paste because its nitrocellulose-based polymeric matrix provides strong adhesion to PET surfaces, rapid curing, and uniform film formation while preserving electrochemical activity^3,35,36^ enabling the development of a PET-based flexible SPE modified with PVP/CuO for the simultaneous determination of DA and PAR. A brief literature summary on disposable sensing platforms for DA and PAR is presented in Table S2.^3,37,38^

PET-based lab-made electrodes offer significant advantages, such as low cost, flexibility, high conductivity, a large active surface area, and reusability through coating with graphite ink. The lightweight, bendable nature of PET makes the electrode particularly suitable for portable, flexible electrochemical sensor applications, while the graphite ink coating increases its conductivity, supporting stable, repeatable signal generation.^3,38,39^ The conductivity of PVP can be improved by doping with conductive materials, particularly metals or metal oxides, thereby establishing a conductive pathway for specific sensing applications.^40^ Among metal oxides, CuO is particularly attractive due to its low cost, water and polymer compatibility, nontoxicity, high physicochemical stability, and catalytic activity arising from abundant active sites;^41^ however, despite the enhanced catalytic performance of copper oxide nanoparticles in sensing and biosensing applications, their tendency to agglomerate can reduce catalytic efficiency, making conductive polymer–metal oxide nanocomposites an effective strategy to increase reactive sites and inhibit aggregation, whereby the PVP-CuO composite deposited by drop-casting enhances both the chemical stability of active sites and the electrocatalytic performance of the electrode while the PVP matrix acts as a protective capping agent that improves the overall stability and performance of CuO nanoparticles.^42,43^ In the literature, no facile, one-step method for preparing polymer–metal oxide films on PET-based lab-made electrodes has been reported.

To the best of our knowledge, this work reports the first PET-based, fully self-fabricated electrode modified with PVP-CuO nanocomposites for the simultaneous detection of DA and PAR. The proposed platform demonstrates that the entire electrode fabrication process can be carried out in a laboratory environment, cost-effectively and in a customizable manner. The integration of recycled PET with conductive nanocomposites provides an environmentally sustainable and mechanically flexible sensing platform, while the PVP-CuO matrix enhances electron transfer and electrochemical performance. The developed sensor demonstrates a wide linear detection range and reliable analytical performance. The results obtained demonstrate the originality of the proposed design and indicate its potential applications in biomedical and pharmaceutical analyses.

## 2 Materials and Methods

### 2.1 Chemicals and Apparatus

PET sheets from used beverage containers were used as substrates, and their surfaces were roughened with 80-grit sandpaper (SAITEX, Italy). Graphite powder (1–5 μm, 99.9%, Nanografi, Türkiye) and conductive silver adhesive ink (EM-Tec AG42, Micro to Nano BV, Netherlands) were used for electrode preparation. A commercial transparent nail polish (Flormar, Türkiye) was used as the polymeric binder. Before modification, PET surfaces were cleaned with Triton X-100 and absolute ethanol. PVP (Mw 360 kDa), CuO nanopowder (<50 nm), paracetamol, dopamine hydrochloride, PBS components, K_4_[Fe­(CN)_6_]·3H_2_O, K_3_[Fe­(CN)_6_], potassium chloride (KCl), potassium hydroxide (KOH), glucose (Glc), ascorbic acid (AA), and dl-aspartic acid (l-Asp), L-3,4-dihydroxyphenylalanine (l-Dopa), (−)-epinephrine (EPI), 5-hydroxytryptamine (Ser) were obtained from Sigma-Aldrich (Germany). Uric acid (UA), urea, l-cysteine (l-cys), creatine (Cr), and l-arginine (l-Arg) were supplied by Fluka (Germany). All chemicals were of analytical grade and applied directly without purification; solutions were prepared using ultrapure water at ambient conditions.

Surface morphology was examined by FE-SEM (QUANTA 400F, USA). FTIR (Shimadzu Infinity, ATR, 4000–600 cm^–1^, Japan) was utilized to analyze the surface functional groups at each modification step. XPS (X-ray Photoelectron Spectroscopy) data were collected using a PHI 5000 VersaProbe instrument (Physical Electronics, USA) with a monochromatic Al Kα source (hν = 1486.6 eV). Contact angle was determined using an Attension Theta instrument (Biolin Scientific, Finland) to assess surface wettability. AFM imaging (NT-MDT Spectrum Instruments, Russia) provided additional topographical information.

### 2.2 Preparation of the Sensor

The preparation of flexible electrochemical sensors fabricated with conductive ink derived from sustainable PET bottles is provided in the Supporting Information (SI) Video M1. Recycled beverage PET sheets were thoroughly cleaned with a nonionic detergent and deionized water, then unidirectionally abraded with 80-grit sandpaper to eliminate the glossy surface and enhance the adhesion of conductive ink. This treatment created a roughened texture, improving the adhesion of the conductive ink.

The FSPE, comprising the working, reference, and counter electrodes, was fabricated according to a layout generated in Silhouette Studio software. The digital pattern was transferred to adhesive sheets using a Silhouette Cameo 4 cutter and then attached to PET substrates as stencils for electrode shaping. Conductive ink, consisting of 40% graphite powder and 60% nail polish, was mechanically mixed for 5 min, then spread across the exposed regions with a spatula to fill the abrasions produced by sanding. After drying, the adhesive masks were peeled away to uncover the electrode areas. Pseudoreference electrodes were prepared by applying silver ink onto designated regions. The completed electrodes were then cut into strips of ∼25 mm × 5 mm to obtain individual FSPEs.

To cure the conductive layer, the electrodes were placed in an oven at 60 °C for 12 h. Electrochemical pretreatment was then carried out by cycling the electrodes in 1.0 M KOH between 0 and 1.5 V for five scans at 50 mV s^–1^.^8^ The PVP-CuO suspension for electrode modification was prepared using an ultrasonic homogenizer (Bandelin Sonopuls, model UW 5050, Germany) operated at 20 kHz frequency and 50 W power with 85% amplitude, employing a pulsed mode (1 min on/1 min off) for a total sonication time of 30 min. This procedure ensured homogeneous dispersion and high colloidal stability of the nanomaterials within the polymeric matrix. Finally, the prepared suspension was applied to the working electrode surface via drop-casting and allowed to dry, yielding PVP-CuO-modified FSPEs, which were ready for electrochemical analysis (Scheme 1).

**Scheme 1:**
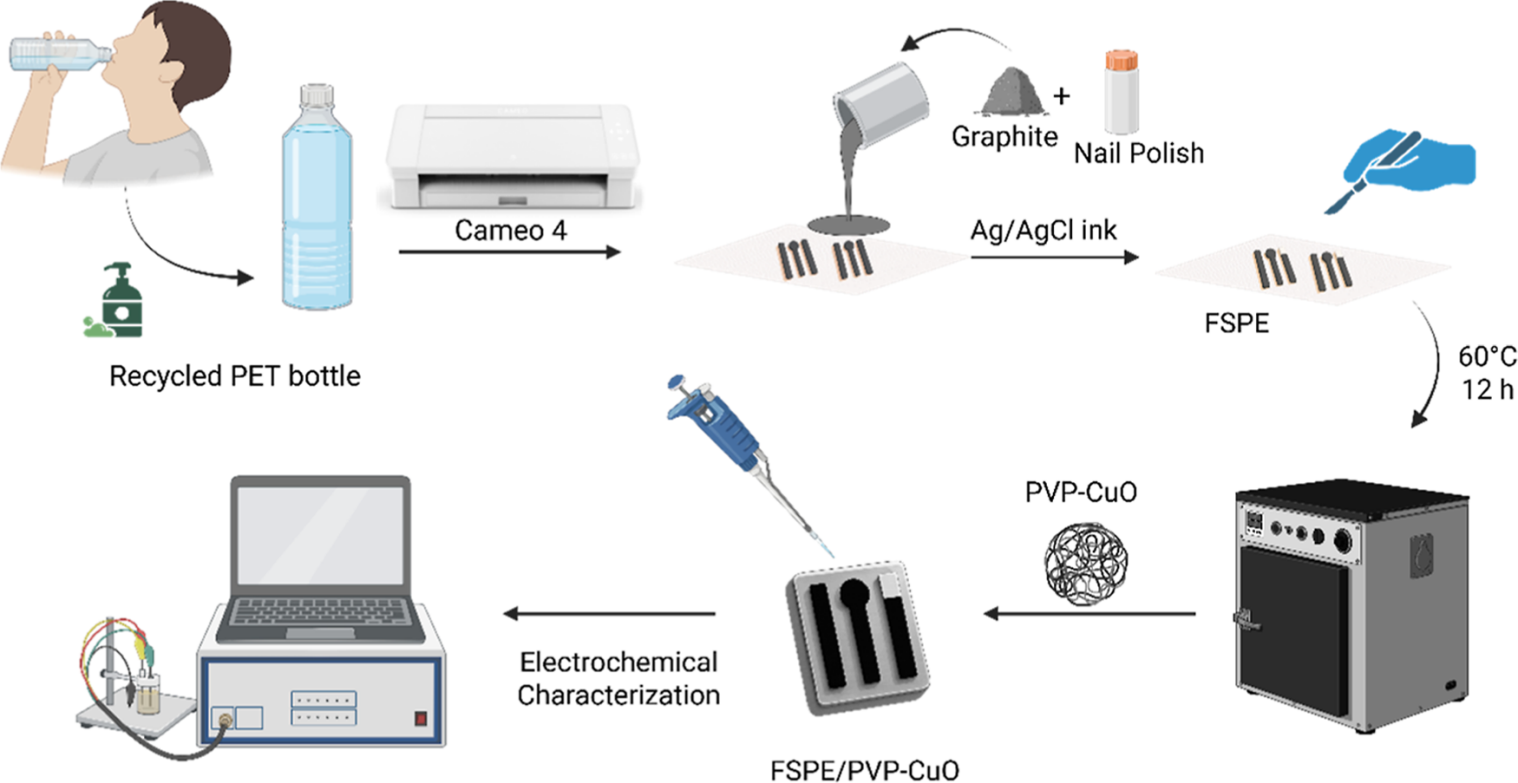
Preparation Steps of the FSPE/PVP-CuO (MFSPEs)^a^

a Created in BioRender. zbitler, D. (2026) https://BioRender.com/14ex8yt.

### 2.3 Electrochemical Measurements

Electrochemical analyses were performed using a PalmSens4 portable potentiostat/galvanostat, operated with PSTrace software. The FSPEs fabricated in this study served as the working electrodes in a three-electrode configuration. Although in many studies researchers need to use external reference and counter electrodes [1] owing to their integrated structure, no external platinum counter or reference electrode was required in this homemade electrode. Electrode modifications were examined through cyclic voltammetry (CV) and differential pulse voltammetry (DPV). CV scans were performed within −0.6 to +1.2 V at a scan rate of 50 mV s^–1^. DPV measurements were recorded in the potential range of −0.2 to +1.0 V under the following conditions: modulation time of 0.02 s, modulation amplitude of 0.05 V, step potential of 0.003 V, and scan rate of 0.015 V s^–1^. For simultaneous detection, DPV was applied to analyze DA and PAR in protonated phosphate-buffered saline (PBS, pH 4.5) over a potential window of −0.2 to +1.0 V. The experiments were carried out at room temperature and replicated three times to ensure reliable results.

### 2.4 Real Sample Analysis

The practical performance of the fabricated FSPE sensor was evaluated using both biological and pharmaceutical samples. For biological validation, fetal bovine serum (FBS, Biological Industries, Haemek, Israel) was diluted 1:10 with PBS and analyzed without further pretreatment. Using DPV and the standard addition method, varying concentrations of DA and paracetamol PAR were spiked to assess simultaneous detection. Commercially available products were used for pharmaceutical application: Dopadren (Vem Pharmaceuticals), a dopamine hydrochloride preparation (200 mg/5 mL), and Parol (Atabay), which contains 500 mg of paracetamol per tablet. For the Dopadren solution, a 0.01 M working solution was prepared directly from the commercial product. For Parol, one tablet was finely ground in a mortar and pestle, suspended in 1000 mL of distilled water, and stirred continuously for 24 h to ensure complete dissolution. The resulting suspensions were filtered, and the filtrates were further diluted to appropriate concentrations falling within the sensor’s calibration range. The prepared drug solutions were analyzed without the addition of external analytes as a standard. DPV measurements were performed on all samples, and percentage recoveries were determined to assess the method’s accuracy.

## 3 Results and Discussion

### 3.1 Surface Characterization of Modified Electrodes

The morphological characteristics of the developed electrodes were evaluated using FTIR spectroscopy to examine the surface chemical properties of FSPE, FSPE/PVP, FSPE/CuO, and the modified FSPE/PVP-CuO, as shown in Figure 1. FTIR spectroscopy is a sensitive and widely used technique for studying chemical interactions and surface modifications. In this study, FTIR spectroscopy was employed to analyze FSPE, composed of graphite integrated with a nail polish binder, together with its stepwise-modified derivatives obtained through PVP coating, CuO coating, and subsequent incorporation of PVP-functionalized CuO, thereby verifying each stage of surface modification as well as the specific contribution of each chemical component. To better understand the FTIR data, the nail polish composition is presented in Table S3. The nail polish used as a binder for integrating the graphite matrix comprises a complex formulation of organic solvents, film-forming agents, plasticizers, polymers, pigments, and fillers.

**Figure 1:**
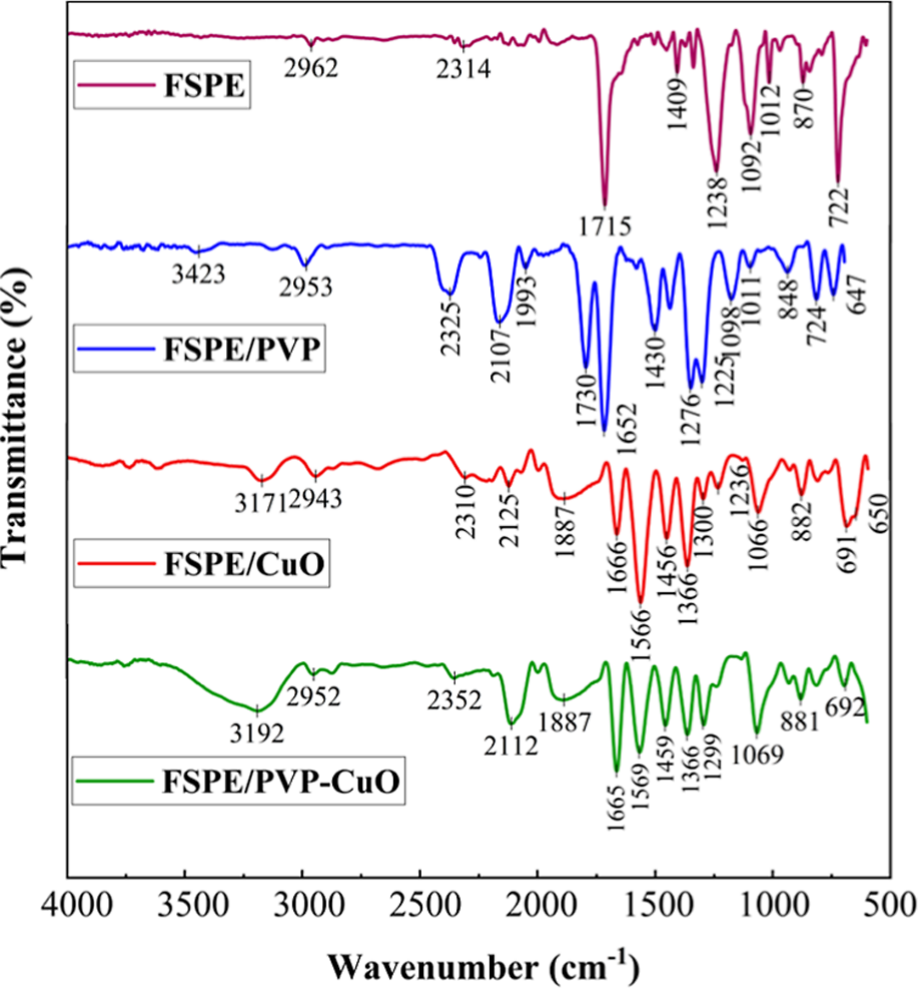
FTIR spectra of FSPE, FSPE/PVP, FSPE/CuO and FSPE/PVP-CuO showing surface chemical modifications.

Additionally, it contains various pigments and colorants, which may contribute to the distinct absorption bands observed in the FTIR spectrum of FSPEs. However, because the electrode surface was exposed to elevated temperatures during curing, a significant portion of the volatile organic compounds likely evaporated into the atmosphere. This thermal evaporation may have led to the attenuation or disappearance of certain characteristic peaks in the FTIR spectrum.

The FTIR spectrum of FSPE displayed several characteristic absorption bands associated with the residual organic constituents of the nail polish binder. A weak band at 2965 cm^–1^ was attributed to aliphatic C–H stretching vibrations originating from hydrocarbon based solvents or plasticizers. The absorption at 2314 cm^–1^ corresponded to atmospheric CO_2_, a common artifact in FTIR measurements. A relatively strong band at 1715 cm^–1^ was assigned to C=O stretching vibrations, most likely arising from ester or ketone functionalities within the nail polish matrix. The absorption band at 1409 cm^–1^ was related to C–H bending or aromatic ring vibrations, with possible contributions from C–O stretching of aromatic esters, alcohols, acid anhydrides, or ketones. The peak at 1238 cm^–1^ was attributed to C–O stretching, typically observed in ester linkages or polymer backbones. Additionally, the bands at 1092, 1012, 870, and 722 cm^–1^ were assigned to aliphatic and aromatic nitro groups, which can be attributed to nitrocellulose and resin components used as film-forming agents in nail polish formulations.^44^

While FSPE exhibits a single carbonyl band at 1715 cm^–1^, the FSPE/PVP spectrum shows a clear splitting into 1730 and 1652 cm^–1^, corresponding to the ester C=O of the binder and the lactam C=O of PVP. The band at 1409 cm^–1^ shifts to 1430 cm^–1^ and increases in intensity upon addition of PVP, reflecting enhanced C–N contributions. New or intensified bands at 1276/1225 cm^–1^ and 1098–1011 cm^–1^ further confirm the presence of PVP through C–N and C–O vibrations.^45,46^ In the higher wavenumber region, a narrow band at 3423 cm^–1^ emerges, in contrast to the broad 3430 cm^–1^ feature reported for hydrated PVP, confirming the dry nature of the coating.^47^ Collectively, these spectral changes demonstrate the successful deposition of PVP on the FSPE surface.

To further highlight the effect of CuO, the FTIR spectrum of FSPE/CuO was recorded. Compared with FSPE, the FSPE/CuO spectrum shows apparent shifts in the carbonyl region, where the binder-related absorption around 1715 cm^–1^ moves toward 1666 cm^–1^, accompanied by new bands at 1566 cm^–1^, 1456 cm^–1^, and 1366 cm^–1^ that reflect interactions between CuO and the organic matrix. In the low-frequency region, strong absorptions at 691 cm^–1^ and 650 cm^–1^ are characteristic of Cu–O stretching vibrations, consistent with the typical behavior of metal oxides below 1000 cm^–1^.^48^ A broad band around 3171 cm^–1^, together with the absorption at 1666 cm^–1^ can be attributed to O–H stretching and H–O–H bending vibrations of adsorbed water molecules.^49^

In the FSPE/PVP-CuO spectrum, a broad absorption at 3192 cm^–1^ is assigned to O–H stretching, while the band at 1665 cm^–1^ corresponds to the lactam C=O stretching of PVP that is shifted due to coordination with CuO. Additional absorptions at 1569 cm^–1^, 1459 cm^–1^, and 1366 cm^–1^ reflect enhanced skeletal C–N and CH_2_ modes of PVP upon coordination. Features at 1299 and 1069 cm^–1^ indicate coupled C–N and C–O vibrations within the coordinated PVP framework. The distinct band near 692 cm^–1^ is characteristic of Cu–O stretching, in agreement with the typical absorptions of metal oxides below 1000 cm^–1^.^48^ These spectral signatures confirm the formation of a coordinated PVP-CuO interface on the graphite/nail polish matrix.

To clarify the chemical composition and oxidation states of the elements present in CuO and PVP-CuO, an XPS analysis was performed in this study. The XPS survey spectra of CuO and PVP-CuO are exhibited in Figure 2A B. As shown in Figure 2B, the additional N 1s and C 1s peaks indicate the formation of PVP-CuO.^50^ The survey spectrum of the nanocomposite shows characteristic peaks from both CuO and PVP, confirming the successful preparation of the composite. The O 1s, Cu 2p, C 1s, and N 1s photoelectrons are displayed in Figure S1, respectively. A positive shift of O 1s photoelectrons (in the CO group of PVP) from 530.32 to 530.90 eV is found in the CuO-PVP sample.^51^ Consistent with these observations, the BE of Cu 2p_3/2_ is shifted negatively from 933.84 eV (pure CuO, Figure S1C) down to 933.65 eV in the CuO-PVP sample (Figure S1D). The transition of the copper ion during photoemission to the |2p53d9> state is represented by the satellites in the Cu 2p spectra.^52^ This figure also clearly shows that Cu 2p_1/2_ and Cu 2p_3/2_ exhibit intense satellites on the high-binding-energy side, separated by approximately 9 eV, which is in good agreement with previous reports.^53^

**Figure 2:**
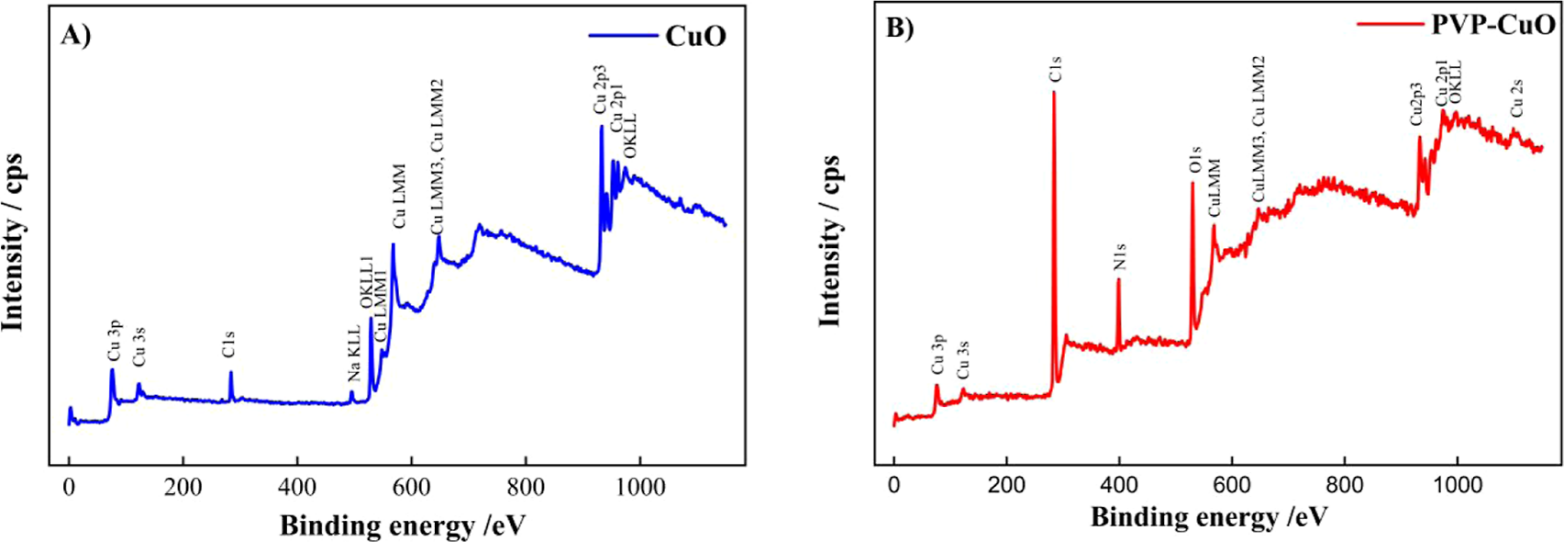
XPS survey spectra for (A) CuO and (B) PVP-CuO.

Similarly, the N 1s shows a positive shift in binding energy to 399.77 eV, compared to that of pure PVP (398.8 eV) as reported in the literature.^54^ All these observations suggest charge transfer between PVP and CuO. Apparently, new energy states may be created by PVP near the conduction band of CuO in these PVP-CuO samples. Indeed, this XPS study also indicates an electronic interaction between the CuO and PVP phases in our samples, supporting the FTIR results.

To complement the morphological analysis, SEM images were taken from the surfaces of FSPE (A), FSPE/PVP (B), and FSPE/PVP-CuO (C) electrodes. Figure 3 shows apparent morphological differences after each surface modification step, while Figure S2 presents the particle-size distribution of the CuO nanostructures. The bare FSPE (A) exhibited a rough surface composed of graphite.^55^ Upon PVP modification (B), the surface appeared smoother and more compact, suggesting the formation of a uniform polymer layer.^56,57^ After CuO nanoparticle loading (C), numerous bright, spherical particles were observed across the surface, confirming the successful deposition of CuO nanoparticles onto the electrode.^40^

**Figure 3:**
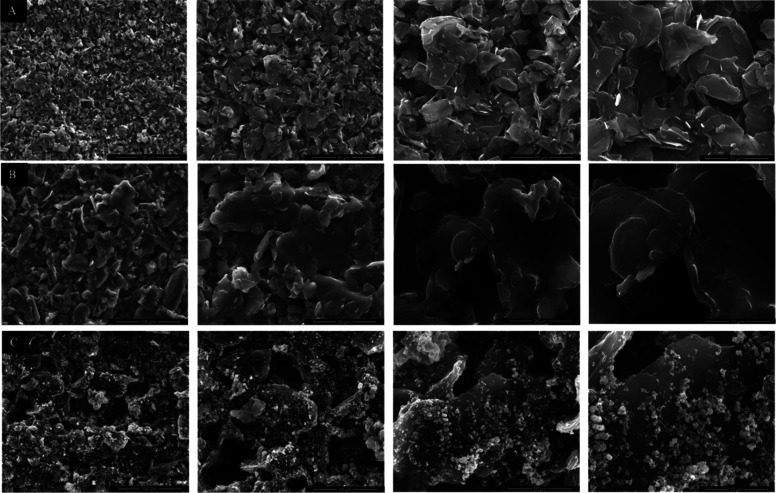
SEM images of (A) FSPE, (B) FSPE/PVP, and (C) FSPE/PVP-CuO electrodes recorded at increasing magnifications (from left to right: 100 μm, 50 μm, 20 μm, 10 μm).

AFM was employed to evaluate the surface morphology and quantify the roughness of both FSPE and the FSPE/PVP-CuO. The 3D AFM in Figure S3 revealed that the bare FSPE exhibited a relatively smooth surface with an average roughness Sa of 58.18 nm and an RMS roughness Sq of 77.08 nm. Upon modification with the PVP-CuO nanocomposite, the surface became more textured due to nanomaterial depositionresulting in increased height variations and more pronounced surface features.^58^

The contact angle analyses were performed for bare and FSPE/PVP-CuO electrodes (Figure 4). The contact angle of the bare FSPE was determined as 58.26°, which is slightly lower than the value reported for pure graphite surfaces (∼68.6°).^59^ This difference can be attributed to the binder phase and the surface roughness of the fabricated FSPE. Upon surface modification, the contact angle decreased to 35.28° for FSPE/PVP and 27.14° for FSPE/PVP-CuO, confirming the enhancement of surface hydrophilicity. These results demonstrate that polymeric coatings and metal oxide nanostructures improve wettability and electrolyte accessibility.

**Figure 4:**
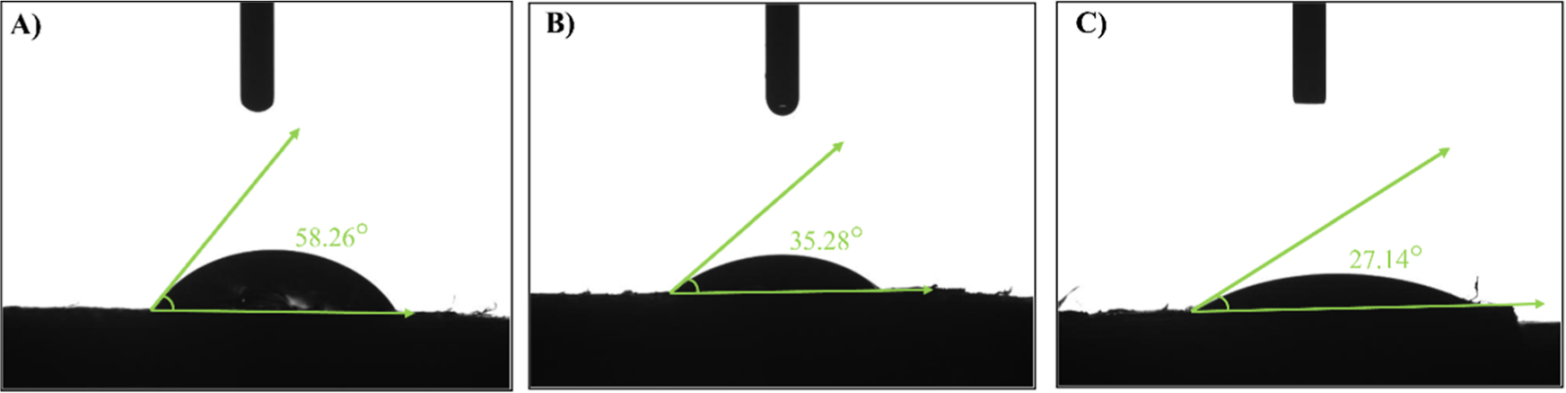
Contact angle measurements of the prepared electrode surfaces: (A) FSPE (58.26°), (B) FSPE/PVP (35.28°), and (C) FSPE/PVP-CuO (27.14°).

### 3.2 Electrochemical Characterization of Modified Electrodes

CV was employed to investigate the electrochemical responses of FSPE, FSPE/PVP, FSPE/CuO, and FSPE/PVP-CuO in 1.0 M KCl containing 5.0 mM [Fe­(CN)_6_]^3–^/^4–^, as shown in Figure 5. Additionally, the effect of varying CuO concentrations on electrode performance was examined. The resulting CVs are shown in Figure S4 for electrodes with CuO loadings of 1, 2, 3, and 4 mg/mL. For the bare FSPE in Figure 5A (curve a), the [Fe­(CN)_6_]^3–/4–^ redox couple exhibited the lowest anodic and cathodic peak currents with a wide peak-to-peak separation (Δ*E*_p_ = 715 mV), confirming sluggish electron-transfer kinetics. After modification with PVP (curve b) the redox peak current decreased, yielding a Δ*E*_p_ of 1157 mV. This indicates that PVP insulation obstructs electron transport between the redox probes and the electrode surface.^60^ Upon incorporation of CuO (curve c) onto FSPE, the redox currents decreased and the Δ*E*_p_ remained relatively large (1171 mV), indicating that CuO alone did not significantly accelerate electron transfer due to the poor conductivity of CuO.^29^ Notably, the FSPE/PVP-CuO electrode (curve d) demonstrated the most favorable behavior, characterized by the highest peak currents, highlighting the synergistic contribution of PVP and CuO in creating a more conductive interface that effectively promotes the electron-transfer kinetics of the [Fe­(CN)_6_]^3–/4–^ redox system. FSPEs generally exhibit higher solution and contact resistances than conventional glassy carbon electrodes, thereby artificially broadening *E*_p_.^61^ Additionally, the [Fe­(CN)_6_]^3–^/^4–^ redox couple is surface-sensitive and may exhibit slow electron-transfer kinetics on unmodified carbon surfaces, further contributing to the significant peak separation. The obtained Δ*E*_p_ value was similar to that previously reported in the literature for PET-based flexible electrodes and other modified carbon systems.^3,61–64^

**Figure 5:**
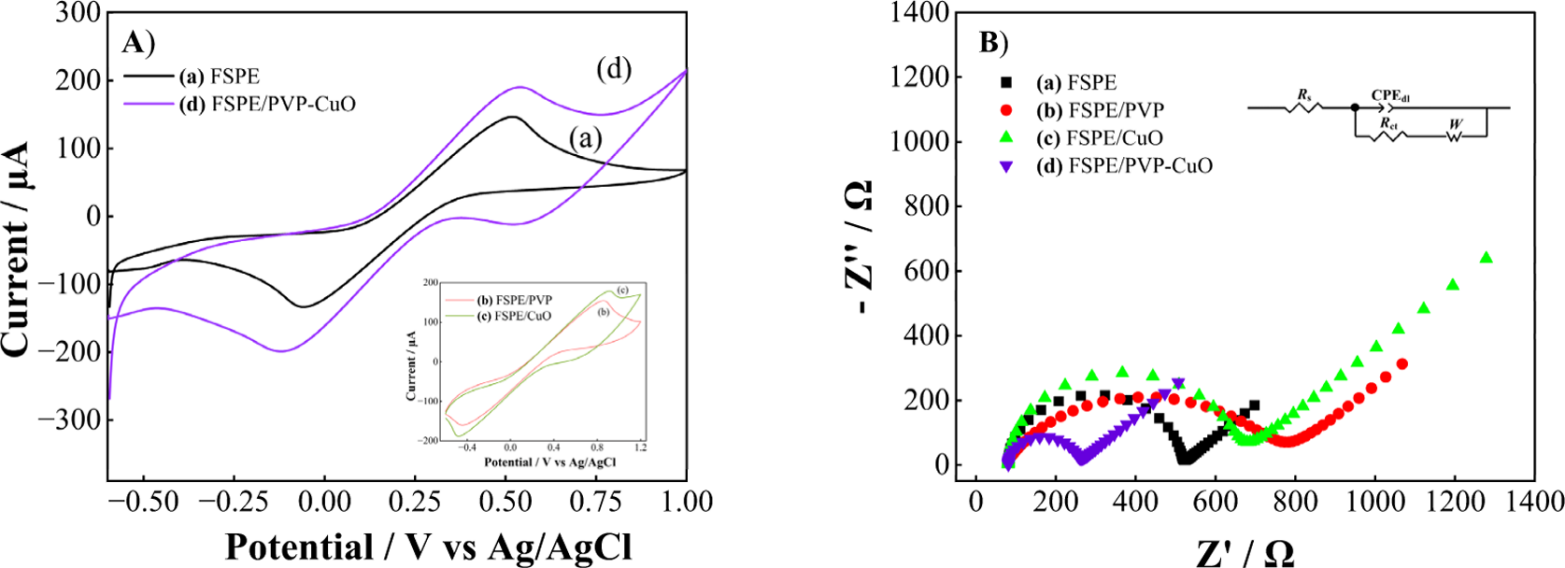
(A) CVs, (B) EIS of (a) FSPE, (b) FSPE/PVP (c) FSPE/CuO (d) FSPE/PVP-CuO electrodes in 1.0 M KCl solution containing 5.0 mM [Fe­(CN)_6_]^3–/4–^.

As shown in Figure 5B, EIS analysis revealed that the FSPE/PVP-CuO electrode exhibited the lowest charge-transfer resistance (171 Ω), which was significantly lower than those of FSPE (433 Ω), FSPE/PVP (689 Ω), and FSPE/CuO (560 Ω). Notably, FSPE/PVP exhibited the highest charge-transfer resistance due to limited electron-transfer kinetics. The combination of PVP with CuO results in a significant reduction, indicating the synergistic improvement in electron-transfer kinetics facilitated by the PVP-CuO nanocomposite. This enhancement is due to PVP’s role in stabilizing and preventing CuO aggregation, thereby increasing the accessibility of redox probes to the electrode surface.^60^ These findings are in good agreement with the CV behavior, which similarly reflects improved electron-transfer characteristics for the FSPE/PVP-CuO electrode.

The voltammetric behavior of the Fe­(CN)_6_^3–/4–^ redox probe was systematically investigated within the potential window of −0.6 to +1.2 V over scan rates from 0.01 to 100 mV s^–1^ using CV. The corresponding CV profiles are displayed in Figure S5. As the scan rate increased, both anodic and cathodic peak currents intensified, accompanied by the expected positive and negative shifts in peak potentials, respectively. To clarify the governing mass-transfer mechanism, the relationship between peak current and the square root of the scan rate was evaluated, and the resulting linear plots indicated diffusion-controlled electrochemical behavior.

The Randles–Ševčík equation is applicable only to systems exhibiting reversible electron transfer; therefore, the reversibility of the modified electrodes was first evaluated by examining the peak-to-peak separation. For a reversible redox couple, Δ*E*_p_ is theoretically ∼59/n mV at 25 °C. Larger values indicate quasi-reversible or irreversible behavior, where Δ*E*_p_ increases and becomes dependent on the scan rate due to kinetic limitations at the electrode interface.^3,65,66^ The method is particularly suitable for quasi-reversible systems, as Δ*E*_p_ increases with scan rate when the mass-transport rate equals or exceeds the electron-transfer rate. Figure S6 displays the *E*_p_ vs ln­(ν) plots, while Figure S7 shows the Δ*E*_p_ (mV) vs scan rate (V s^–1^) curves for both FSPE and FSPE/PVP-CuO electrodes. As illustrated in Figure S7, Δ*E*_p_ increases with increasing scan rate, indicating that the mass-transport rate approaches or surpasses the electron-transfer rate, a defining characteristic of quasi-reversible kinetics.^67^ The PVP-CuO modification exhibits a higher peak current, indicating enhanced mass-transfer efficiency. The linear trends observed in Figure S6 further confirm the applicability of the Laviron approach. Using Laviron analysis, the electron-transfer coefficient (α) and the heterogeneous electron-transfer rate constant (*k* ^0^) were determined from the slopes and intercepts of the corresponding linear plots. According to established electrochemical benchmarks, systems are classified as reversible (*k* ^0^ > 2 × 10^–2^ cm s^–1^), quasi-reversible (*k* ^0^ from 2 × 10^–2^ to 3 × 10^–5^ cm s^–1^), or irreversible (*k* ^0^ < 3 × 10^–5^ cm s^–1^). The calculated k^0^ values, 1.08 × 10^–4^ cm s^–1^ for FSPE and 1.46 × 10^–4^ cm s^–1^ for FSPE/PVP-CuO, confirm that both platforms operate within the quasi-reversible system.^68^ In parallel, α increases by ∼1.35-fold, indicating a lower energetic barrier to charge transfer at the electrode interface. Significantly, the PVP-CuO modification increases the heterogeneous rate constant by approximately 1.35-fold, demonstrating a significant enhancement in interfacial electron-transfer kinetics. This improvement directly reflects the electrocatalytic action of CuO nanostructures, which facilitate faster charge transfer by providing additional active sites, improved conductivity, and more efficient electron-hopping pathways at the electrode surface.^3,65,68^ These results are consistent with the literature, which reports similar *k* ^0^ values for comparable modified electrodes.^67,69^

### 3.3 Electrochemical Performance of DA and PAR at the Bare and Modified Sensors

Electrochemical responses of bare and modified FSPEs for DA and PAR were investigated using CV and DPV. The CV and DPV responses of DA and PAR were studied separately in 0.1 M PBS (pH 4.5) using FSPE, FSPE/PVP, FSPE/CuO, and FSPE/PVP-CuO. Figure 6A,B display the representative CVs, and the corresponding DPV profiles are provided in Figure 6C. As illustrated in Figure 6D, DA and PAR undergo electrochemical oxidation via a two-proton and two-electron transfer process. In this mechanism, the catechol moiety of DA undergoes oxidation to form *o*-quinone, while the phenolic group of PAR is oxidized to *p*-quinone imine.^17,70,71^

**Figure 6:**
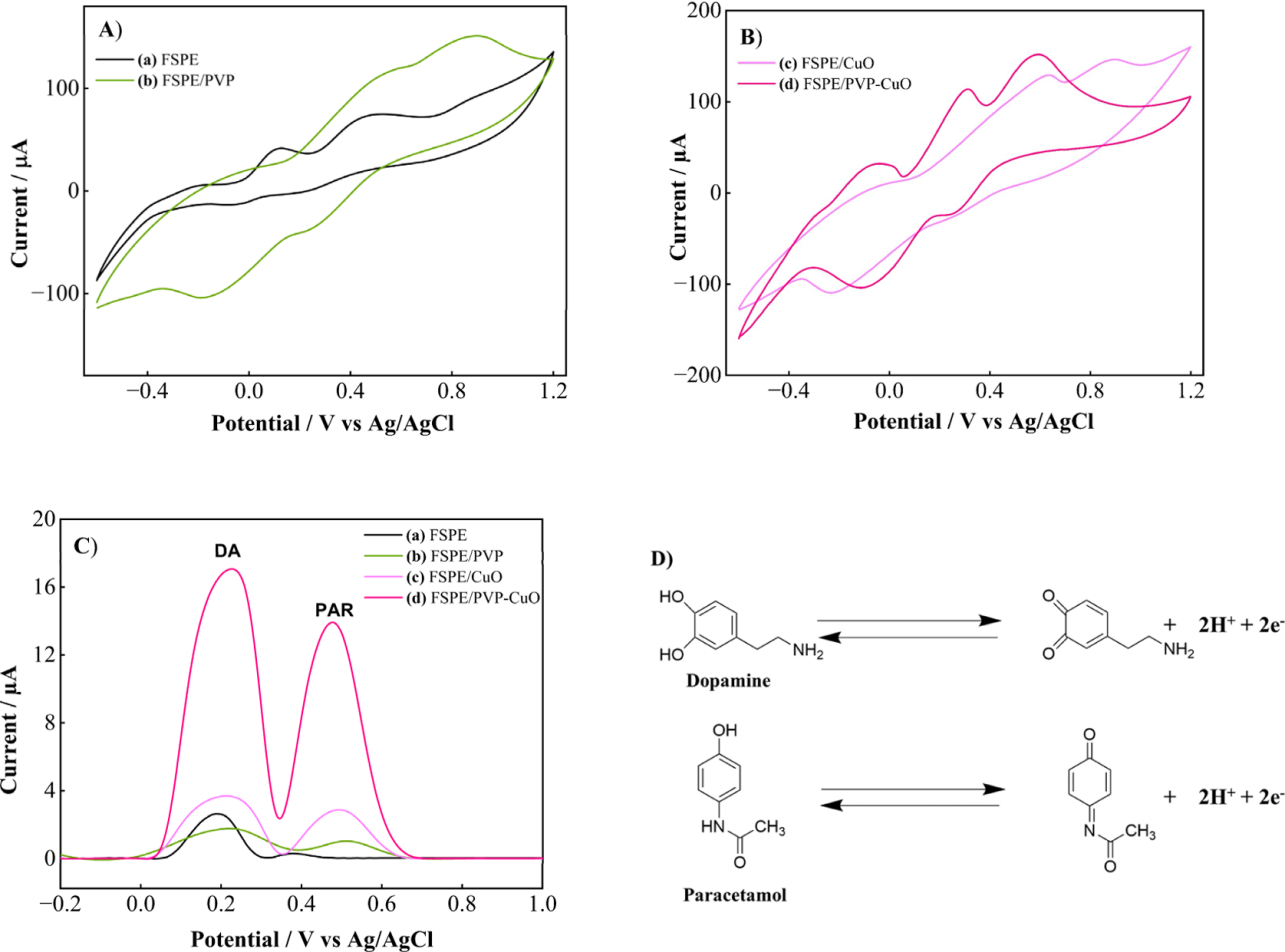
(A) and (B) Comparative CV, (C) DPV responses of 1000.0 μM DA and 1000.0 μM PAR at FSPE electrodes: (a) FSPE, (b) FSPE/PVP, (c) FSPE/CuO, and (d) FSPE/PVP-CuO in 0.1 M PBS (pH 4.5). (D) Electrochemical oxidation mechanisms of DA and PAR at FSPE/PVP-CuO modified electrode.

When the electrodes were tested in a mixed solution of 1000.0 μM DA and 1000.0 μM PAR, distinct differences at the cyclic voltammograms were observed. As shown in Figure 6A,B, FSPE (curve a), FSPE/PVP (curve b), and FSPE/CuO (curve c) displayed broad and weak oxidation signals, where the peaks of DA and PAR could not be effectively distinguished. In contrast, FSPE/PVP-CuO (curve d) produced two sharp and well-separated oxidation peaks at 0.31 and 0.58 V, confirming its excellent selectivity and sensitivity. Among all electrodes, FSPE/PVP-CuO exhibited the highest anodic peak currents and the lowest oxidation potentials, highlighting its superior electrocatalytic activity. This behavior indicates that the modified electrode surface provides more accessible electroactive sites for DA and PAR, thereby strengthening their interaction with the electrode interface. These results clearly demonstrate that the synergistic interaction of PVP and CuO strongly promotes the electrochemical oxidation of DA and PAR by accelerating charge-transfer kinetics. In addition, the improved surface characteristics facilitate the adsorption and efficient oxidation of the analytes, which directly contributes to the enhanced sensitivity of the sensor toward DA and PAR.

DPV analysis (Figure 6C) further confirmed the superior performance of FSPE/PVP-CuO (curve d), which showed the highest current response and two well separated oxidation peaks for DA (0.22 V) and PAR (0.47 V). In contrast, FSPE (curve a), FSPE/PVP (curve b), and FSPE/CuO (curve c) showed relatively weak signals at the oxidation peaks of DA (0.19 V, 0.22 V, 0.21 V) and PAR (0.37 V, 0.52 V, 0.49 V), respectively. As shown in Figure 6B, the peaks were poorly resolved, resulting in significant overlap. In DPV measurements, FSPE/PVP-CuO also provided the clearest signal resolution, enabling well-resolved peaks for DA and PAR, whereas the other electrodes showed partial overlap. This clear peak separation confirms that the PVP-CuO nanocomposite enhances the affinity of the electrode surface toward both analytes. Such improvement arises from the synergistic effect of PVP and CuO nanoparticles, which provide abundant electroactive sites and facilitate faster electron-transfer kinetics, thereby enabling more sensitive and selective electrochemical detection. Overall, these results demonstrate that FSPE/PVP-CuO offers both high sensitivity and clear peak resolution, making it a promising platform for the simultaneous electrochemical detection of DA and PAR.

Figure S8 shows the plots of peak current versus scan rate for 10.0 μM DA and 10.0 μM PAR at the FSPE/PVP-CuO electrode in 0.1 M PBS (pH 4.5). The peak currents increased with increasing scan rate, indicating that the electrochemical responses were dependent on the scan rate. The peak potentials of DA and PAR shifted slightly with increasing scan rate (Figure S8A,B). As shown in the figures, good linear relationships were observed between the peak current and the scan rate for DA with the equations *I*_pc_ (μA) = 109.970*x* (V s^–1^) – 0.226 (*R* ^2^ = 0.994) for the cathodic peak current and *I*_pa_ (μA) = 133.200*x* (V s^–1^) + 4.667 (*R* ^2^ = 0.984) for the anodic peak current. For PAR, only the anodic peak showed a linear relationship with scan rate, with the equation *I*_pa_ (μA) = 138.249*x* (V s^–1^) – 8.109. These results suggest that the electro-oxidation of DA and PAR at the FSPE/PVP-CuO electrode is mainly diffusion-controlled.^72^

### 3.4 Effect of pH

The pH of the supporting electrolyte plays a critical role in determining the electrochemical behavior of DA and PAR. The influence of pH on the current responses and peak potentials is illustrated in Figure 7. As shown in Figure 7A, the DPV voltammograms of DA and PAR recorded at different pH values (3.0–7.0) revealed that both peak positions and current intensities were strongly pH-dependent. At lower pH values, the oxidation peaks appeared at more positive potentials with relatively weak currents, whereas increasing the pH gradually shifted the peaks toward lower potentials and altered the signal intensities.

**Figure 7:**
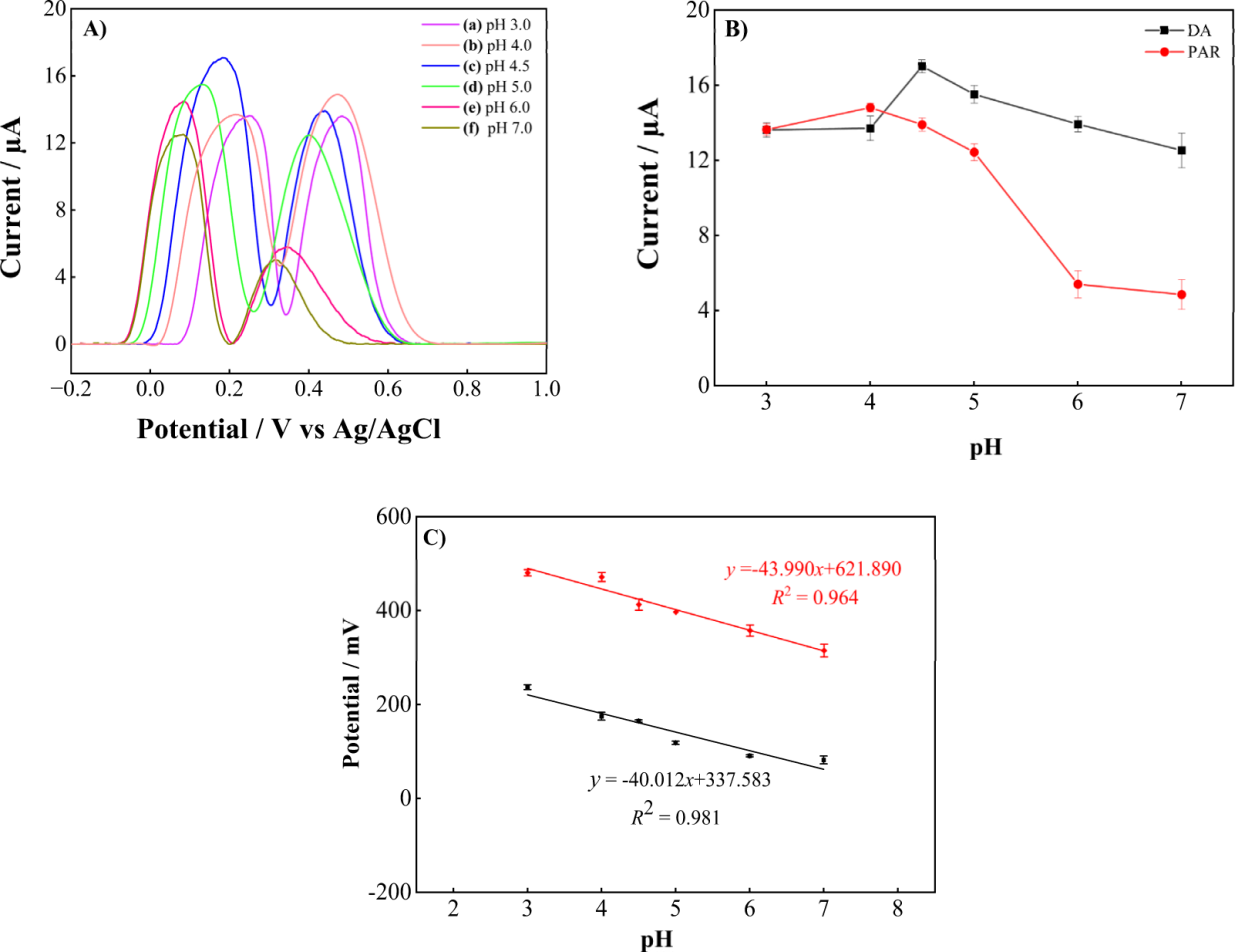
Effect of pH on the electrochemical response of 1000.0 μM DA and 1000.0 μM PAR in 0.1 M PBS (pH 3.0–7.0): (A) DPV voltammograms at different pH values, (B) variation of DPV peak currents, and (C) variation of DPV peak potentials.

As shown in Figure 7B, the oxidation currents of both DA and PAR reached their maximum at pH 4.5, beyond which the currents decreased as the medium became less acidic. Therefore, pH 4.5 was chosen as the optimum pH for subsequent analyses, and similar results were obtained in studies in the literature.^73–75^ In addition, the oxidation peak potentials systematically shifted toward more negative values with increasing pH (Figure 7C), confirming that the electrochemical processes were proton-coupled. The corresponding linear regression equations were calculated as *E*_pa_ = −40.01 pH + 337.583 (*R* ^2^ = 0.981) for DA and *E*_pa_ = −43.990 pH + 621.890 (*R* ^2^ = 0.964) for PAR. The slopes obtained for DA (−40.01 mV pH^–1^) and PAR (−43.990 mV pH^–1^) are close to the value of the theoretical Nernstian slope, 59.0 mV pH^–1^, indicating that the redox reactions involve an equal number of electrons and protons.^76,77^

### 3.5 Individual and Simultaneous Voltammetric Determination of DA and PAR

The corresponding DPVs for the individual detection of DA and PAR are presented in Figure 8A–D. As shown in Figure 8A–D, the analyte concentrations increased gradually over the studied range. The fabricated sensor exhibited wide dynamic detection ranges for DA and PAR, from 1.0 to 1000.0 μM. The linear regression equations were determined as *I*_DA_ (μA) = 0.013*C*_DA_ (μM) – 0.070 (*R* ^2^ = 0.998) and *I*_PAR_ (μA) = 0.010*C*_PAR_ (μM) – 0.227 (*R* ^2^ = 0.996) for DA and PAR, respectively. The limit of detection (LOD) and limit of quantification (LOQ) were estimated using the expressions 3 s/m and 10 s/m, respectively, where *s* represents the standard deviation of the analytical response and *m* denotes the slope of the calibration curve. For individual analysis, the LOD values were estimated at 0.310 μM for DA and 0.0902 μM for PAR, whereas the LOQ values were calculated at 0.941 μM and 0.301 μM for DA and PAR, respectively.

**Figure 8:**
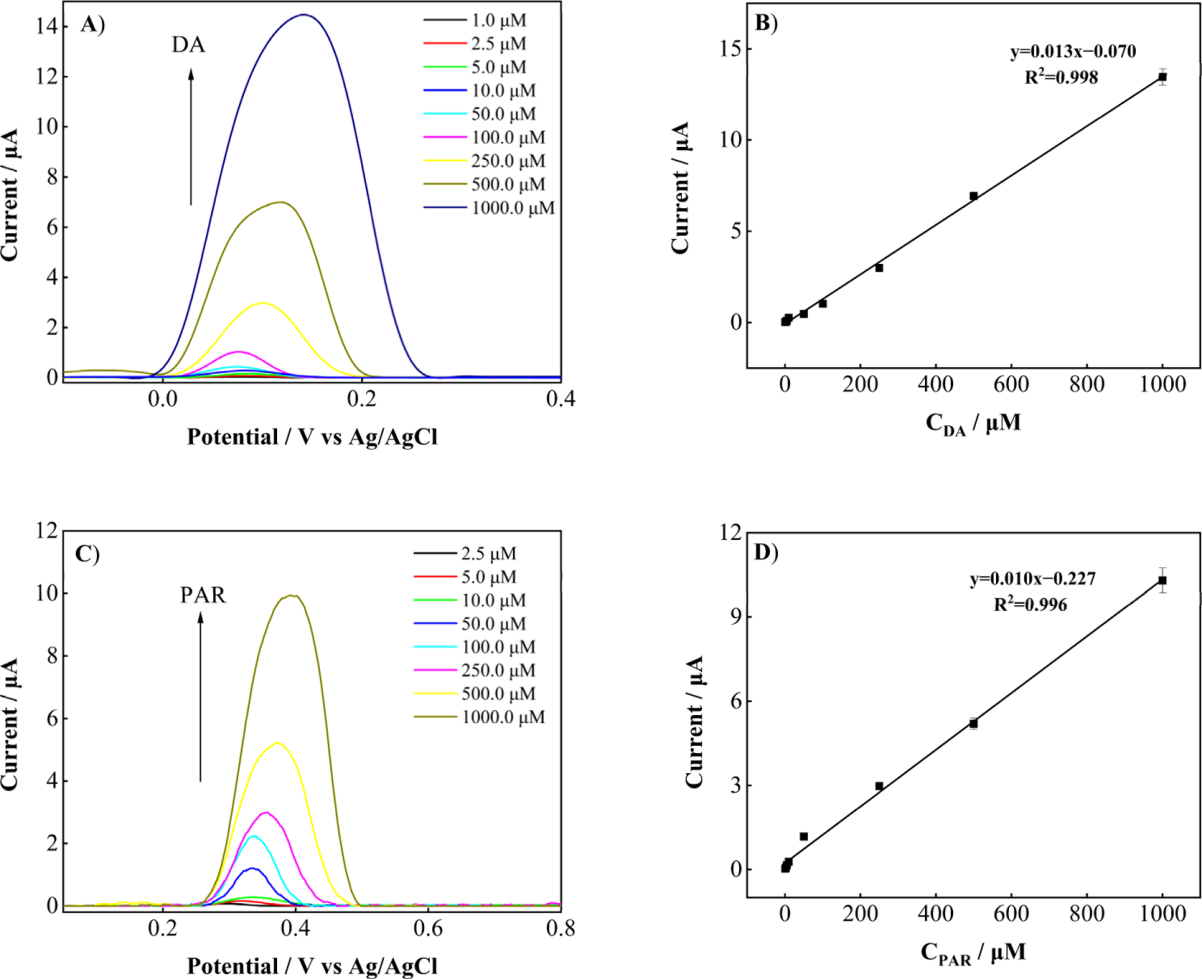
DPVs obtained at the FSPE/PVP-CuO electrode in 0.1 M PBS (pH 4.5): (A) DA in the concentration range of 1.0–1000 μM, (B) corresponding calibration plot for DA, (C) PAR in the concentration range of 1.0–1000 μM, and (D) corresponding calibration plot for PAR.

Furthermore, the simultaneous determination of DA and PAR was examined in their mixed solution by DPV, as shown in Figure 9A. Two well-separated anodic peaks (for DA at 0.22 V and for PAR at 0.47 V) gradually increased with increasing analyte concentration (Figure 9B,C). The linear working ranges were 4.0–1000.0 μM for DA (*R* ^2^ = 0.992) and PAR (*R* ^2^ = 0.995). The calculated LOD values were 1.188 μM for DA and 1.024 μM for PAR, while the LOQ values were 3.960 μM and 3.415 μM, respectively. Standard error of slope and standard error of intercept information are also included in Table S4. These findings demonstrate that DA and PAR can be simultaneously detected with high selectivity at the FSPE/PVP-CuO electrode. This enhanced activity is due to the synergistic effect of PVP-CuO, which provides high catalytic activity and excellent stability.

**Figure 9:**
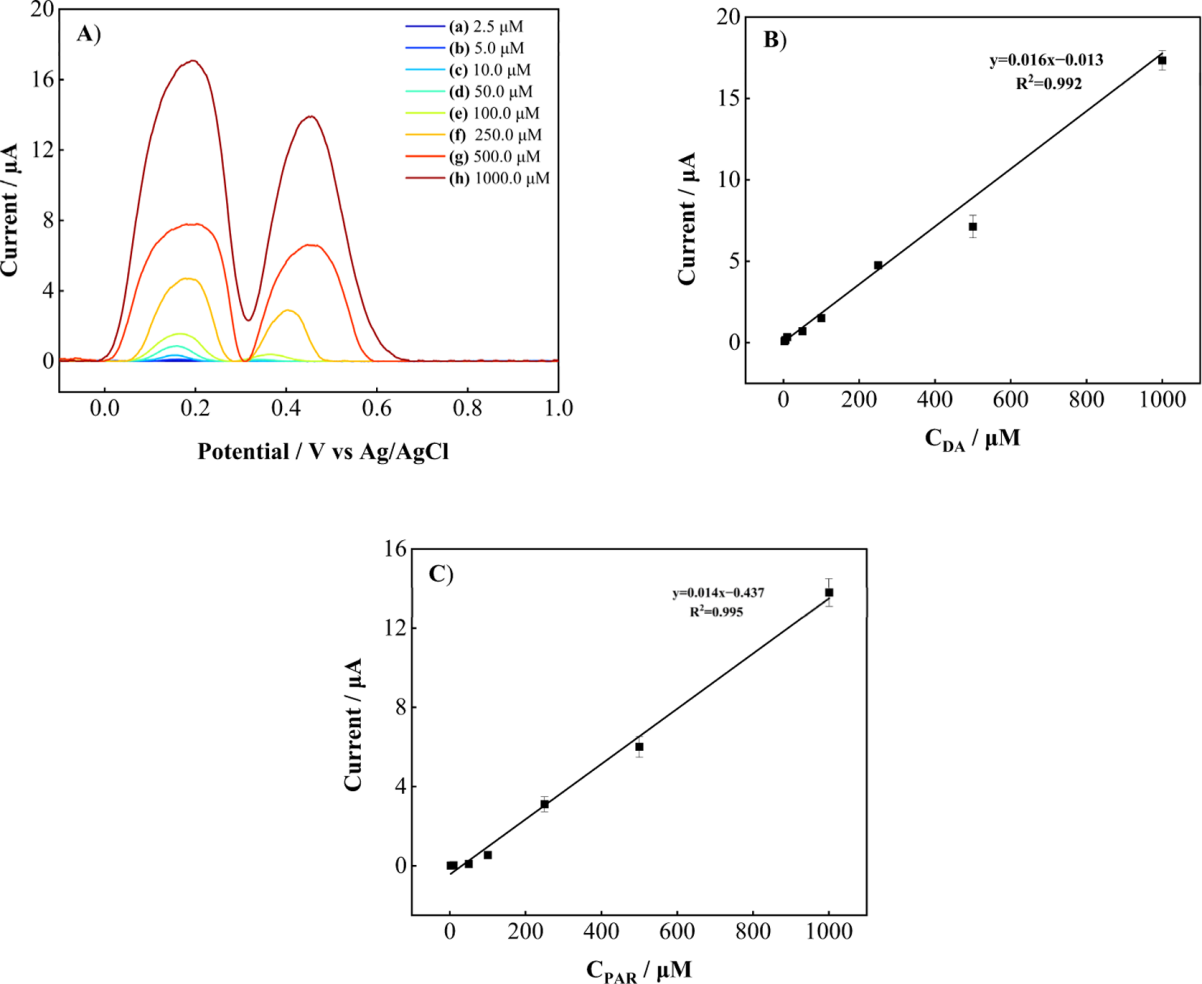
(A) DPVs for the simultaneous detection of DA and PAR at the FSPE/PVP-CuO electrode in 0.1 M PBS (pH 4.5) and (B) the corresponding calibration plots for DA and (C) PAR (*n* = 3).

### 3.6 Interference Study

To evaluate the selectivity of the fabricated lab-made electrode, potential interfering compounds such as urea, Glc, AA, l-Cys, l-Arg, l-Asp, Cr, l-Dopa EPI, UA, and Ser were examined by recording the responses of 100.0 μM DA and 100.0 μM PAR in the presence of 100.0 μM of various coexisting species in 0.1 M PBS (pH 4.5). Three DPV measurements were made for every interference parameter, and the average peak currents were examined. As shown in Figure 10, in the presence of these interference compounds, the DA and PAR exhibit current responses ranging from 94.59% to 103.84%, and these molecules cause interference effects of less than ±10% for both analytes, demonstrating that the modified electrode exhibits satisfactory selectivity toward the target analytes.

**Figure 10:**
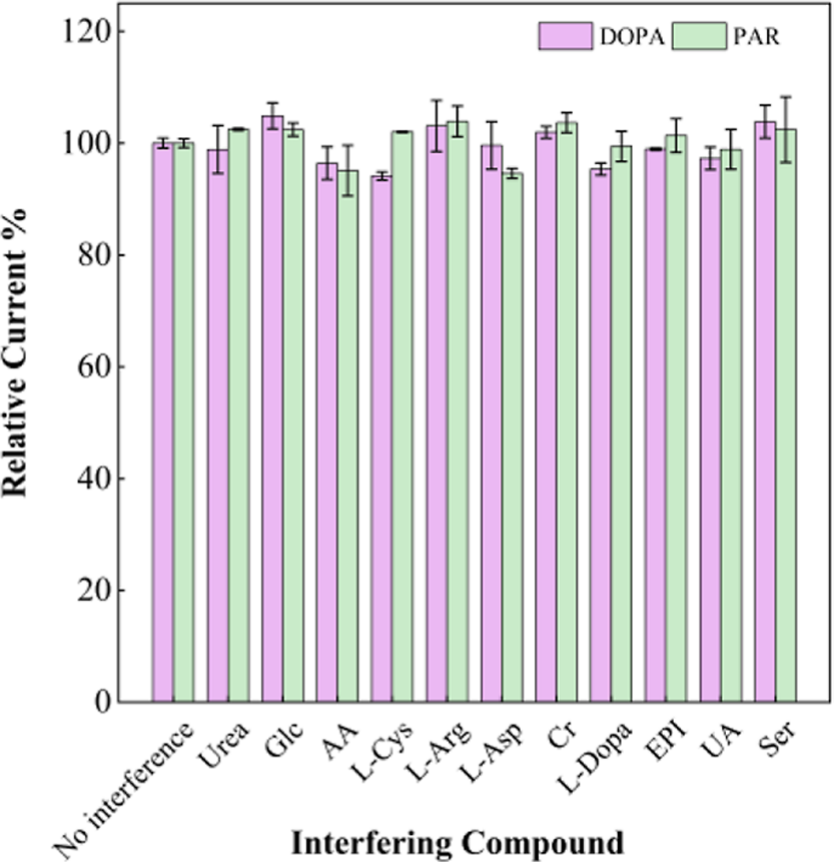
DPV peak currents of the FSPE/PVP-CuO for 100.0 μM DA, and 100.0 μM PAR in 0.1 M PBS (pH 4.5) before (DA and PAR) and after adding 100 μM Urea, Glc, AA, l-Cys, l-Arg, l-Asp, Cr, l-Dopa EPI, UA and Ser.

### 3.7 Repeatability, Reproducibility, and Storage Stability

The analytical reliability of the developed electrode was systematically examined through repeatability, reproducibility, and storage stability tests. For repeatability, five consecutive measurements of the target analytes (100.0 μM) were recorded under identical conditions, yielding relative standard deviation (RSD) values of less than 5%, indicating highly consistent responses. The reproducibility assessment using three independently fabricated electrodes showed consistent responses, with all RSD % values below 10%. Finally, the electrode’s storage stability was evaluated by storing it at ambient laboratory conditions (25 °C) for 7 days. After this period, the electrode maintained 85% of its initial response to DA and 80% to PAR, confirming satisfactory stability at room temperature. The results are summarized in Table 1.

**Table 1:** Analytical Performance of the Developed Electrode in Terms of Repeatability, Reproducibility, and Storage Stability

parameter	experimental condition	performance (RSD %/retained signal %)
Repeatability	5 sequential measurements (100.0 μM analyte, same day)	3.65% (DA), 4.57% (PAR)
Reproducibility	3 independent electrodes (100.0 μM analyte)	5.5% (DA), 5.4% (PAR)
Storage stability	7 days at +25 °C	85% (DA), 80% (PAR)

### 3.8 Real Sample Analysis

To evaluate the practical feasibility of the proposed electrode, analyses were performed on commercial drug formulations-Dopadren (containing dopamine, DA) and Parol (containing paracetamol, PAR)-as well as on commercial fetal bovine serum to assess the simultaneous detection of DA and PAR. Before spiking, the baseline concentrations of DA and PAR in each real sample were measured, and all recovery values were calculated after adjusting for this initial content to ensure accurate quantification. The results obtained (Table 2) showed recoveries ranging from 100% to 108% for dopamine and from 94% to 105% for paracetamol. These values fall within the commonly accepted range for pharmaceutical assays (90–110%), confirming the accuracy of the sensing platform. Furthermore, the relative standard deviations remained consistently low (<5%), indicating high precision even within complex serum matrices. The corresponding current responses of DA and PAR obtained from the pharmaceutical samples and FBS are presented in Figure S9. Overall, these findings verify the developed sensor’s ability to accurately, precisely, and reliably quantify DA and PAR in both pharmaceutical formulations and serum samples.

**Table 2:** Simultaneous Determination of DA and PAR in Dopadren, Parol, and Commercial Fetal Bovine Serum Using the Developed Electrode, Showing Satisfactory Recoveries (SD, *n* = 3) and Acceptable Precision

sample	analyte	added (μM)	found (μM)	recovery (%)	RSD (%)
Dopadren	Dopamine	10.0	10.02	100.24 (±0.011)	4.06
		50.0	50.69	101.39 (±0.021)	4.96
		100.0	107.60	107.60 (±0.012)	1.13
Parol	Paracetamol	10.0	9.44	94.40 (±0.013)	5.08
		50.0	48.33	96.66 (±0.056)	4.87
		100.0	105.38	105.38 (±0.035)	1.50
FBS	Dopamine	10.0	10.48	104.84 (±0.015)	4.33
		50.0	50.32	100.64 (±0.022)	3.96
		100.0	98.89	98.89 (±0.042)	4.02
	Paracetamol	10.0	10.15	101.48 (±0.001)	3.89
		50.0	52.49	104.98 (±0.001)	4.77
		100.0	98.38	98.38 (±0.019)	0.87

## 4 Conclusion

A disposable, flexible and lab-made electrochemical sensing platform was innovatively engineered through the direct transfer of a low-cost, graphite-based conductive ink onto recycled PET substrates, followed by the uniform drop casting of a PVP-CuO nanocomposite prepared via ultrasonication. The resulting FSPE/PVP-CuO electrode exhibited excellent structural uniformity, mechanical robustness, and enhanced electrochemical activity. The synergistic contribution of the PVP-CuO modification further facilitated rapid and efficient charge transport throughout the conductive network. The sensor exhibited outstanding analytical performance for both individual and simultaneous determination of DA and PAR. In the independent mode, broad linear ranges (1.0–1000.0 μM) were achieved with low LOD/LOQ values of 0.310/0.941 μM for DA and 0.0902/0.301 μM for PAR. Under simultaneous detection conditions, the sensor maintained high sensitivity, providing linear ranges of 4.0–1000.0 μM with LOD/LOQ values of 1.188/3.960 μM for DA and 1.024/3.415 μM for PAR. Interference studies verified its strong selectivity in the presence of common coexisting species (urea, Glc, AA, l-Cys, l-Arg, l-Asp, Cr), while real-sample analyses confirmed its high accuracy and reliability across pharmaceutical matrices and biological fluids. This could significantly aid in the development of a low-cost transducer for the practical simultaneous quantification of DA and PAR. Although this study produced encouraging results, there are still potential drawbacks regarding the adaptability of the connectors and cables used for commercial SPE. To enhance the adaptability of the flexible, lab-made electrode for a range of real-world applications, research should also investigate a multianalyte detection strategy. Finally, the proposed FSPE/PVP-CuO system combines affordability, versatility, and environmental sustainability, positioning it as a powerful next-generation platform for practical and high-performance electrochemical sensing applications.

## Supplementary Material





## Notes

Figures for XPS high resolution spectrum for CuO, PVP-CuO, histogram of CuO particle size distribution extracted from SEM micrographs, 3D AFM images, CVs of electrodes prepared with varying CuO concentrations, plot of the peak current against the square root of the scan rate, Ep vs ln v plot, peak current vs the scan rate plot. Tables for the cost analysis of the materials used for the fabrication, Summary of recent studies on disposable electrochemical sensors, chemical composition of the nail polish, Electrochemical characteristics for bare and modified electrodes, standard error of slope and standard error of intercept information (PDF)
SI Video last.mp4 (MP4)
