# Supplementary material for: Fabrication of a Flexible Lab-Made PVP-CuO Modified Electrochemical Sensor for Dual Detection of Dopamine and Paracetamol
Source: ACS Omega. 2026 Apr 27;11(18):27216–29. doi: 10.1021/acsomega.6c01227 (PMC13177042; doi:10.1021/acsomega.6c01227)
Supplement: Supplementary file 1 [file ao6c01227_si_001.pdf]

Supplementary Information

Fabrication of a Flexible Lab-Made PVP-CuO Modified Electrochemical Sensor for Rapid Dual Detection of Dopamine and Paracetamol

Duygu ZABİTLER[1,2], Esra ÜLKER[1], Gözde AYDOĞDU TIĞ[2*]

[1]Ankara University Graduate School of Natural and Applied Sciences

[2]Ankara University, Faculty of Science, Department of Chemistry, Ankara, 06100, Türkiye

*Corresponding author: Gözde AYDOĞDU TIĞ, gaydogdu@science.ankara.edu.tr

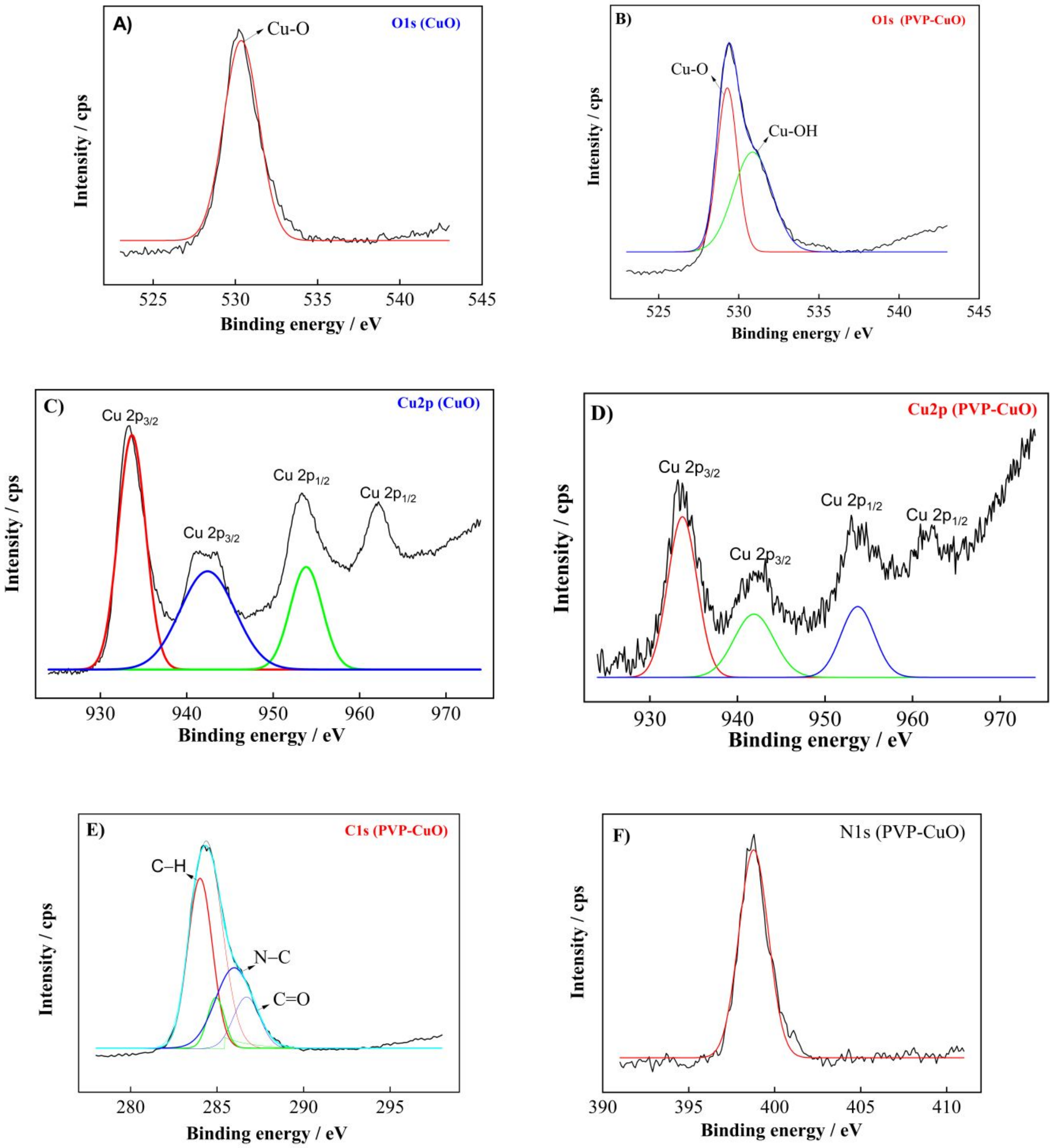

Figure S1. XPS high-resolution spectrum for CuO (A, C), PVP-CuO (B, D, E, F)

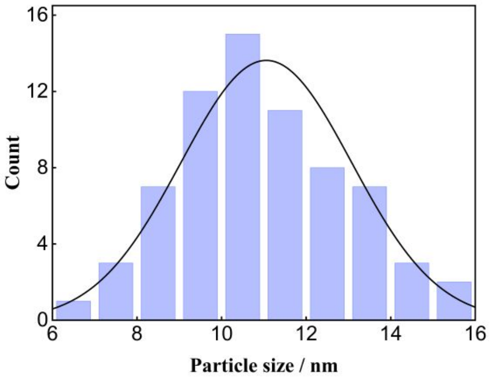

Figure S2. Histogram of CuO particle size distribution extracted from SEM micrographs.

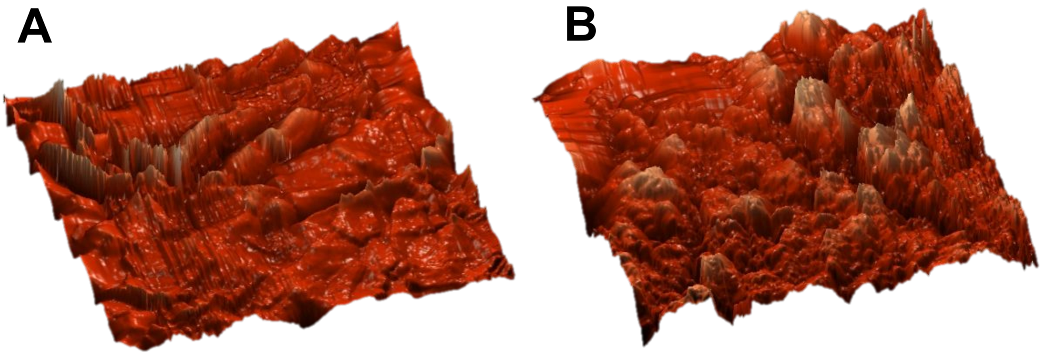

Figure S3. 3D AFM images of (A) FSPE and (B) FSPE/PVP-CuO (5 × 5 μm²).

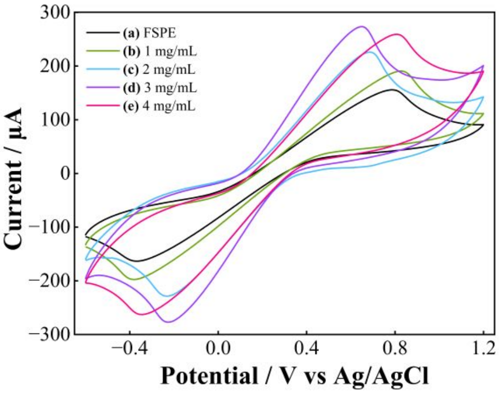

Figure S4. CVs of electrodes prepared with varying CuO concentrations: (a) FSPE, (b) 1 mg/mL, (c) 2 mg/mL, (d) 3 mg/mL, and (e) 4 mg/mL.

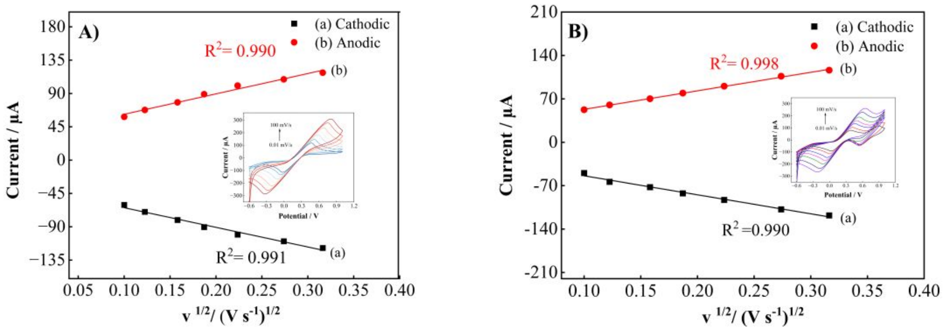

Figure S5. The plot of the peak current against the square root of the scan rate at the (A) FSPE and (B) FSPE/PVP-CuO electrodes in 1.0 M KCl solution containing 5.0 mM $Fe(CN)_6^{3-/4-}$ (inset graphs show CV responses of the electrode at different scan rates).

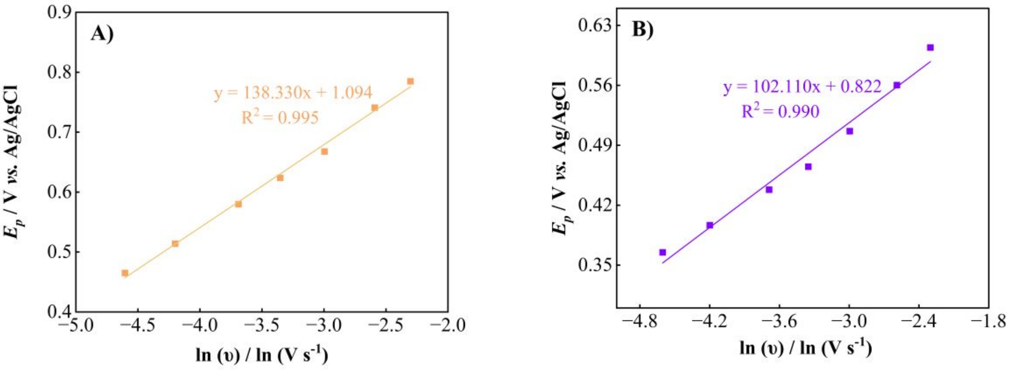

Figure S6. E_p *vs*. ln *v* plot for (A) FSPE, (B) FSPE/PVP-CuO electrodes in 1.0 M KCl solution containing 5.0 mM $Fe(CN)_6^{3-/4-}$.

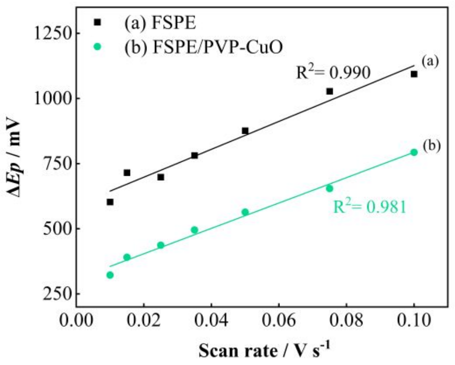

Figure S7. Peak current vs. the scan rate plot for (a) FSPE and (b) FSPE/PVP-CuO in 1.0 M KCl solution containing 5.0 mM $Fe(CN)_6^{3-/4-}$.

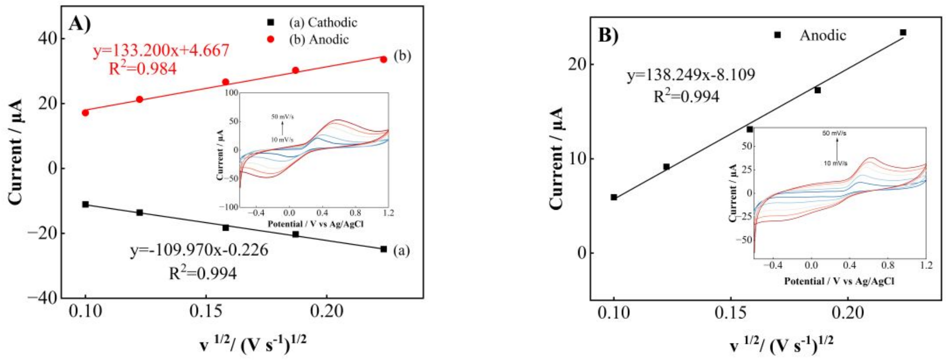

Figure S8. Plots of peak current vs. scan rate for 10.0 µM (A) DA and 10.0 µM (B) PAR at the FSPE/PVP-CuO electrode in 0.1 M PBS (pH 4.5); inset plots show the corresponding CV responses at scan rates ranging from 10–50 mV s^{-1}.

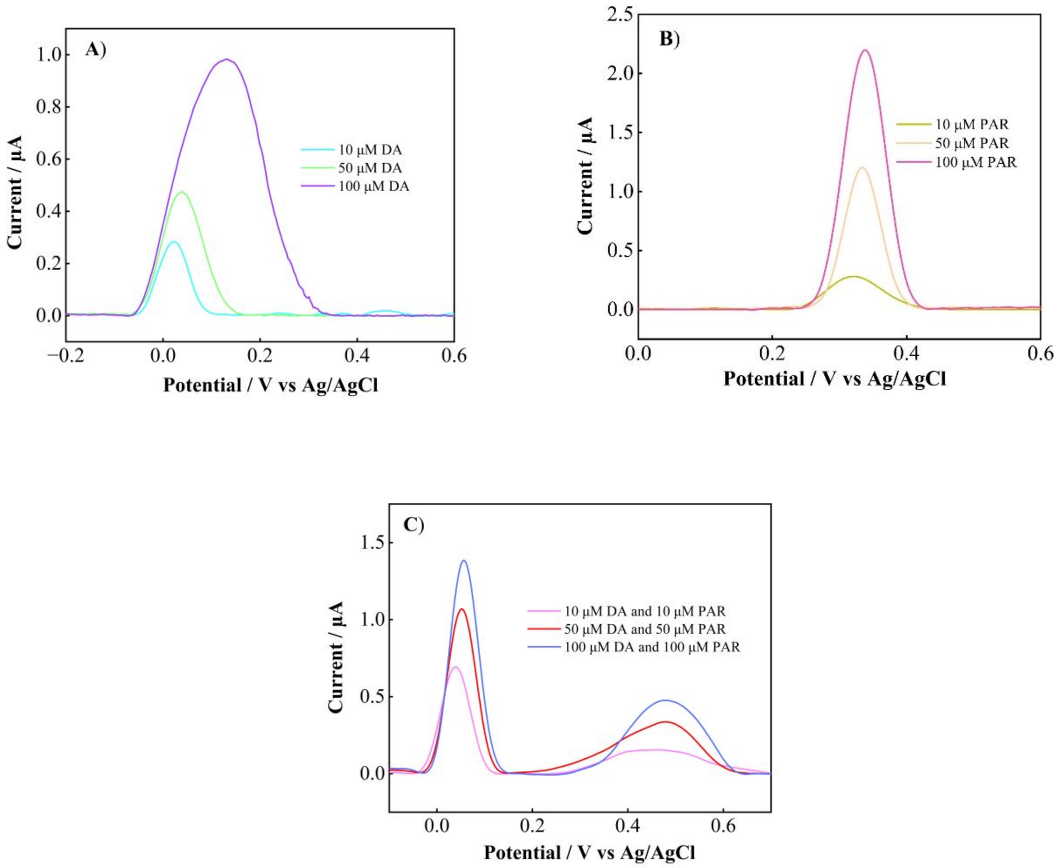

Figure S9. DPV responses for the detection of (A) DA in Dopadren, (B) PAR in Parol, and (C) simultaneous detection of DA and PAR in FBS.

Table S1. Cost analysis of the materials used for the fabrication of 100 lab-made MFSPEs.

Material	Unit price (USD)	Amount used	Total cost (USD)
Graphite	1.750/g	12 g	21.00
Silver Ink	7.733/g	2 g	15.47
Nail Polish	0.160/g	40 g	6.40
Adhesive paper	2.78 (per sheet)	1 sheet	2.78
CuO	0.002/g	3 g	0.006
PVP	0.0003/g	3 g	0.0009
**Total MFSPE cost**	**0.4565 USD per electrode**	**100 electrode**	**45.65**

Table S2. Summary of recent studies on disposable electrochemical sensors for DA and PAR, including analytical performance parameters reported in the literature.

Sensor Platform	Method	Linear Range	LOD	Ref
SPCE/CB-ERGO	DPV	DA: 4.9 – 19 µM<br>PAR: 9.9 – 95 µM<br>EP: 9.9 – 95 µM	DA: 1.50 µM<br>PAR: 9.40 µM<br>EP: 5.30 µM	[36]
NCQDs/ITO-PET	CV	DA: 0 – 2000 µM	DA: 4.7 µM	[37]
PT-FSPE	DPV	PAR and CAF: 10.0–2000.0 µM	PAR: 6.15 µM<br>CAF: 3.16 µM	[3]
FSPE	DPV	DA and PAR: 2.5 – 1000 µM	DA: 1.13 µM<br>PAR: 0.98 µM	This study

Table S3. Chemical composition of the nail polish used in the fabrication of FSPE.

Nail Polish Ingredients
The nail polish used as a binder contains a complex formulation of organic solvents, film-forming agents, plasticizers, polymers, pigments, and fillers. Its composition includes butyl

acetate, ethyl acetate, nitrocellulose, adipic acid/neopentyl glycol/trimellitic anhydride copolymer, acetyl tributyl citrate, isopropyl alcohol, styrene/acrylates copolymer, acrylates copolymer, stearalkonium bentonite, n-butyl alcohol, silica, benzophenone-1, trimethylpentanediyl dibenzoate, titanium dioxide [nano], polyvinyl butyral, synthetic fluorphlogopite, phthalic anhydride/trimellitic anhydride/glycols copolymer, MEK, stearalkonium hectorite, benzophenone-3, aluminium hydroxide, methicone, tin oxide, and calcium aluminum borosilicate. It may also contain various pigments and colorants such as CI 77891 (titanium dioxide), CI 15850 (Red 6, 7, and 34 Lake), CI 77491 (iron oxides), CI 19140 (Yellow 5 Lake), CI 77266 [nano] (Black 2), CI 47005 (Yellow 10 Lake), CI 77510 (ferric ammonium ferrocyanide), CI 77007 (ultramarines), CI 77163 (bismuth oxychloride), CI 77000 (aluminum powder), and CI 60725 (Violet 2).

Table S4. Standard error of slope and standard error of intercept information

**Analyte**	**Standard Error of Slope**	**Standard Error of Intercept**
DA (individual)	2.20×10^{-4}	0.085
PAR (individual)	2.54×10^{-4}	0.103
DA (simultaneous)	6.20×10^{-4}	0.252
PAR (simultaneous)	3.79×10^{-4}	0.261
